# Supplementary material for: Resolving the full spectrum of human genome variation using Linked-Reads
Source: Genome Res. 2019 Apr;29(4):635–45. doi: 10.1101/gr.234443.118 (PMC6442396; doi:10.1101/gr.234443.118)
Supplement: Supplemental Material [file supp_gr.234443.118_Supplemental_longranger-2.2.zip › longranger-2.2.tar.bz2!/lr2.2-public/lib/stan/cmdstan/stan/doc/api/html/agrad_2rev_2functions_2multiply__log_8hpp_source.html]

Stan: src/stan/agrad/rev/functions/multiply\_log.hpp Source File


|  |  |
| --- | --- |
|  | Stan  2.5.0  probability, sampling & optimization |


- Main Page
- Related Pages
- Namespaces
- Classes
- Files

- File List
- File Members

All Classes Namespaces Files Functions Variables Typedefs Enumerations Enumerator Friends Macros Pages

- src
- stan
- agrad
- rev
- functions

multiply\_log.hpp

Go to the documentation of this file.

1 #ifndef STAN\_\_AGRAD\_\_REV\_\_FUNCTIONS\_\_MULTIPLY\_LOG\_HPP

2 #define STAN\_\_AGRAD\_\_REV\_\_FUNCTIONS\_\_MULTIPLY\_LOG\_HPP

3

4 #include <limits>

5 #include <stan/agrad/rev/var.hpp>

6 #include <stan/agrad/rev/internal/vv\_vari.hpp>

7 #include <stan/agrad/rev/internal/vd\_vari.hpp>

8 #include <stan/agrad/rev/internal/dv\_vari.hpp>

9 #include <stan/agrad/rev/functions/log.hpp>

10 #include <stan/math/functions/multiply\_log.hpp>

11 #include <boost/math/special\_functions/fpclassify.hpp>

12

13 namespace stan {

14  namespace agrad {

15

16  namespace {

17  class multiply\_log\_vv\_vari : public op\_vv\_vari {

18  public:

19  multiply\_log\_vv\_vari(vari\* avi, vari\* bvi) :

20  op\_vv\_vari(stan::math::multiply\_log(avi->val\_,bvi->val\_),avi,bvi) {

21  }

22  void chain() {

23  using std::log;

24  if (unlikely(boost::math::isnan(avi\_->val\_)

25  || boost::math::isnan(bvi\_->val\_))) {

26  avi\_->adj\_ = std::numeric\_limits<double>::quiet\_NaN();

27  bvi\_->adj\_ = std::numeric\_limits<double>::quiet\_NaN();

28  } else {

29  avi\_->adj\_ += adj\_ \* log(bvi\_->val\_);

30  if (bvi\_->val\_ == 0.0 && avi\_->val\_ == 0)

31  bvi\_->adj\_ += adj\_ \* std::numeric\_limits<double>::infinity();

32  else

33  bvi\_->adj\_ += adj\_ \* avi\_->val\_ / bvi\_->val\_;

34  }

35  }

36  };

37  class multiply\_log\_vd\_vari : public op\_vd\_vari {

38  public:

39  multiply\_log\_vd\_vari(vari\* avi, double b) :

40  op\_vd\_vari(stan::math::multiply\_log(avi->val\_,b),avi,b) {

41  }

42  void chain() {

43  using std::log;

44  if (unlikely(boost::math::isnan(avi\_->val\_)

45  || boost::math::isnan(bd\_)))

46  avi\_->adj\_ = std::numeric\_limits<double>::quiet\_NaN();

47  else

48  avi\_->adj\_ += adj\_ \* log(bd\_);

49  }

50  };

51  class multiply\_log\_dv\_vari : public op\_dv\_vari {

52  public:

53  multiply\_log\_dv\_vari(double a, vari\* bvi) :

54  op\_dv\_vari(stan::math::multiply\_log(a,bvi->val\_),a,bvi) {

55  }

56  void chain() {

57  if (bvi\_->val\_ == 0.0 && ad\_ == 0.0)

58  bvi\_->adj\_ += adj\_ \* std::numeric\_limits<double>::infinity();

59  else

60  bvi\_->adj\_ += adj\_ \* ad\_ / bvi\_->val\_;

61  }

62  };

63  }

64

77  inline var multiply\_log(const var& a, const var& b) {

78  return var(new multiply\_log\_vv\_vari(a.vi\_,b.vi\_));

79  }

90  inline var multiply\_log(const var& a, const double b) {

91  return var(new multiply\_log\_vd\_vari(a.vi\_,b));

92  }

104  inline var multiply\_log(const double a, const var& b) {

105  if (a == 1.0)

106  return log(b);

107  return var(new multiply\_log\_dv\_vari(a,b.vi\_));

108  }

109

110  }

111 }

112 #endif

boost::math::isnan

bool isnan(const stan::agrad::var &v)

Checks if the given number is NaN.

**Definition:** boost\_fpclassify.hpp:69

multiply\_log.hpp

dv\_vari.hpp

log.hpp

vd\_vari.hpp

stan::agrad::var::vi\_

vari \* vi\_

Pointer to the implementation of this variable.

**Definition:** var.hpp:40

unlikely

#define unlikely(x)

**Definition:** likely.hpp:9

var.hpp

stan::agrad::var

Independent (input) and dependent (output) variables for gradients.

**Definition:** var.hpp:27

stan::agrad::multiply\_log

fvar< T > multiply\_log(const fvar< T > &x1, const fvar< T > &x2)

**Definition:** multiply\_log.hpp:15

vv\_vari.hpp

stan::agrad::log

fvar< T > log(const fvar< T > &x)

**Definition:** log.hpp:15


---

[ Stan Home Page ]

*© 2011–2014,
Stan Development Team.*
